# Recent Advances in Cryo Electron Microscopy and Tomography

**DOI:** 10.1063/4.0001009

**Published:** 2025-10-27

**Authors:** Marc M. H. Storms, Eliza Nieweglowska, Matt Joens

**Affiliations:** 1 Thermo Fisher Scientific, Eindhoven, Noord Brabant, Netherlands; 2 Thermo Fisher Scientific, Los Angeles, California, USA

## Abstract

In the recent years, cryo-electron microscopy (cryo-EM) single-particle analysis (SPA) has greatly benefited from a multitude of technological advances cumulating in breaking the atomic-resolution barrier for biological macromolecules. Since then, structural insights by cryo-EM have led to driving rational design of new vaccines and therapeutics due to high throughput and high-resolution screening and data collection technologies. Additionally, cryo-electron tomography (cryo-ET) has become an indispensable tool for structural cell biology. In this presentation we elaborate on the latest Thermo Fisher Scientific innovations for 300kV cryo electron microscopy for SPA and cryo-ET to advance more workflow efficiency and performance. Our ongoing mission is to further improve the connectivity between the cryo-FIB and cryo-TEM for an even more streamlined tomography workflow that is safer, faster and cleaner. Data acquisition and processing can be a significant cryo-ET bottleneck. Our integrated solution, combining Thermo Scientific Tomography 5 and Tomo Live, automates pre-acquisition tasks, enhances productivity with predictive tracking and enables rapid quality assessment. Robust and optimized motion correction helps produce crisp tomograms and image quality needed for sub-tomogram averaging. Results are displayed with new quality metrics on a web platform, making the workflow user-friendly and facilitating structural biology advancements.

